# Dissipation, Processing Factors and Dietary Risk Assessment for Flupyradifurone Residues in Ginseng

**DOI:** 10.3390/molecules27175473

**Published:** 2022-08-26

**Authors:** Nan Fang, Changpeng Zhang, Zhongbin Lu, Zhou Lu, Zhongbei Zhang, Bo Wang, Zhiguang Hou, Xueping Zhao

**Affiliations:** 1 College of Plant Protection, Jilin Agricultural University, Changchun 130118, China; 2 State Key Laboratory for Managing Biotic and Chemical Threats to the Quality and Safety of Agro-Products, Ministry of Agriculture and Rural Affairs Key Laboratory for Pesticide Residue Detection, Institute of Agro-Products Safety and Nutrition, Zhejiang Academy of Agricultural Sciences, Hangzhou 310021, China; 3 Laboratory of Quality & Safety Risk Assessment for Ginseng and Antler Products, Jilin Agricultural University, Changchun 130118, China; 4 Agricultural Product Quality Inspection and Monitoring Center, Baishan Municipal Bureau of Agriculture and Rural Affairs, Baishan 134399, China

**Keywords:** flupyradifurone, ginseng, analytical method, mass spectrometry, pesticide residue analysis

## Abstract

The massive use of pesticides has brought great risks to food and environmental safety. It is necessary to develop reliable analytical methods and evaluate risks through monitoring studies. Here, a method was used for the simultaneous determination of flupyradifurone (FPF) and its two metabolites in fresh ginseng, dried ginseng, ginseng plants, and soil. The method exhibited good accuracy (recoveries of 72.8–97.5%) and precision (relative standard deviations of 1.1–8.5%). The field experiments demonstrated that FPF had half-lives of 4.5–7.9 d and 10.0–16.9 d in ginseng plants and soil, respectively. The concentrations of total terminal residues in soil, ginseng plants, dried ginseng, and ginseng were less than 0.516, 2.623, 2.363, and 0.641 mg/kg, respectively. Based on these results, the soil environmental risk assessment shows that the environmental risk of FPF to soil organisms is acceptable. The processing factors for FPF residues in ginseng were 3.82–4.59, indicating that the concentration of residues increased in ginseng after drying. A dietary risk assessment showed that the risk of FPF residues from long-term and short-term dietary exposures to global consumers were 0.1–0.4% and 12.07–13.16%, respectively, indicating that the application of FPF to ginseng at the recommended dose does not pose a significant risk to consumers.

## 1. Introduction

Ginseng (*Panax ginseng* C. A. Mey.) is one of the most commonly used ginseng botanicals in the world, mainly consumed in fresh and processed form. Because of the special cultivation environment (loose and fertile brown forest soil with a deep humus layer and high-water content), insect pests and fungal diseases are the biggest problems that affect ginseng cultivation [1]. Pesticide application is essential to ensure high yield and quality of ginseng but also causes environmental and food safety problems. Therefore, the residue analysis and risk assessment of pesticides in ginseng and its products are very important.

Flupyradifurone (FPF, Figure S1) is the first representative of the novel butenolide class of insecticides developed by Bayer [2]. It is effective on the pests resistant to neonicotinoid insecticides and has less adverse effects on honeybee colonies [3,4,5]. FPF has been applied to many agricultural and horticultural crops such as apples, cotton, rice, tomatoes, potatoes, and berries (strawberries, blackberries, and raspberries), and has been registered in the US, EU, and Australia [6,7]. The maximum residue limits (MRLs) of FPF for crops in these countries are 0.01–3 mg/kg. China has set MRLs for FPF in some crops in 2021 [8]. Difluoroacetic acid (DFA) and 6-chloronicotinic acid (6-CNA) were the main metabolites of FPF [9]. DFA was high leachability, very mobile, moderate aquatic ecotoxicology, and moderate mammals acute toxicity, and 6-CNA was moderately mobile, moderate aquatic ecotoxicology, and low mammals acute toxicity [10]. Therefore, the residue definition of FPF was the sum of FPF, DFA, and 6-CNA, and expressed as FPF [9], and the determination of DFA and 6-CNA were important.

Currently, there are few studies on the dissipation, terminal residues, processing factors (PFs), and dietary risk assessment of FPF in food and agricultural products [11]. Most of the related data for FPF comes from the Bayer and Joint FAO/WHO Meeting on Pesticide Residues (JMPR). Li et al. [12] developed a method for the determination of FPF and two other metabolites in fruits, vegetables, and grains. However, relevant data on FPF in ginseng and its products (dried ginseng) have not yet been reported. Therefore, the aims of this study were to (a) determine the residue levels of FPF and its metabolites in ginseng (fleshy taproot) and processed commodities (dried ginseng); (b) evaluate the dissipation of FPF and its metabolites in ginseng plants (the part of the stem and leaves on the ground) and soil, and (c) assess the dietary risk to consumers. 

## 2. Results and Discussion

### 2.1. Optimization of Sample Preparation

**Figure 1:**
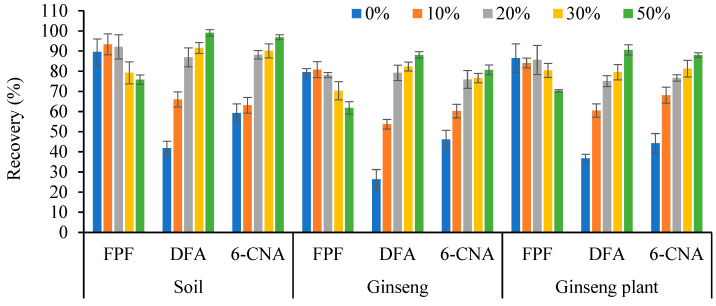
Recovery of flupyradifurone, difluoroacetic acid, and 6-chloronicotinic acid in soil, fresh ginseng, and ginseng plants for the method using different proportions of water (0–50%) in the extraction solution.

It was reported that 6-CNA and DFA were difficult to extract in water using the QuEChERS method [13] because of their octanol–water partition coefficients (Log P of the 6-CNA and DFA was 0.98 and −0.11, respectively) [10]. We also found that the extraction efficiencies of DFA and 6-CNA improved significantly upon the addition of formic acid, and the recoveries did not increase when the concentration of formic acid reached 2%. Thus, a mixture of acetonitrile and water containing 2% formic acid was used for extraction, and the water content (0–50%) in the mixture was studied. The results demonstrate that as the content of water in the extraction solution increased, the FPF recovery decreased and was less than 70% when the content of water was 50% (Figure 1). However, the recoveries of DFA and 6-CNA increased significantly. Satisfactory recoveries of the three compounds were obtained when the water:acetonitrile ratio in the extraction solution was 1:4 (*v*/*v*). 

Primary secondary amine (PSA) has been proven to have a strong adsorption effect on 6-CNA and DFA, and the purification effect of Sorbents octadecyl silica (C_18_) and graphitised carbon black (GCB) in several common sorbents was enhanced [12,13]. It is probably due to the presence of two amino groups in PSA, which leads to a strong adsorption to polar compounds. Therefore, C_18_ was used for the purification of soil and ginseng (the recoveries were 73.4–96.7%), and a combination of C_18_ and GCB was used for the purification of ginseng plants (the recoveries were 72.8–94.8%) in this study (Table S3).

### 2.2. Validation Results of Analytical Method

The mean recoveries of FPF, 6-CNA, and DFA from each sample spiked at all levels were 72.8–97.5%, with intraday and interday relative standard deviations (RSDs) of 1.1–5.7% and 3.3–8.5%, respectively (Table S3). The limit of quantitation (LOQs) for FPF and 6-CNA as per the developed method were 0.01 mg/kg, and that for DFA was 0.05 mg/kg in each matrix. Good linearity (R^2^ = 0.9991–0.9999) was observed from all matrix-matched calibration curves (Table S4).

### 2.3. Dissipation of FPF and Its Metabolites in Ginseng Plants, Soil, and Ginseng

**Figure 2:**
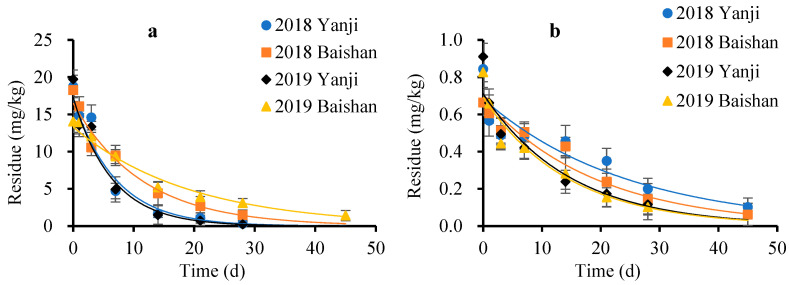
Degradation kinetic curve of flupyradifurone in ginseng plants and soil: (**a**) ginseng plants; (**b**) soil.

The validated method was successfully applied to the determination of analytes in soil and ginseng plant samples in the dissipation experiment. The results demonstrate that the dissipation of FPF in soil and fresh ginseng plants followed a first-order kinetic model (Figure 2 and Table 1). 

**Figure 3:**
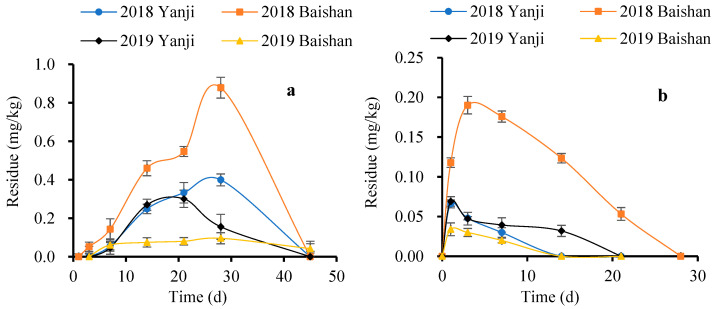
Change of concentration of difluoroacetic acid and 6-chloronicotinic acid in ginseng plants: (**a**) difluoroacetic acid; (**b**) 6-chloronicotinic acid.

The initial deposits of FPF in ginseng plants were 14.03–19.27 mg/kg. The residue of FPF in ginseng plants decreased by 77.82–98.78% to 0.24–3.11 mg/kg on day 28. The calculated half-lives of FPF in ginseng plants were 4.5–7.9 d, indicating that FPF is an easily degradable pesticide in ginseng plants (t_1/2_ < 30 d), and the different climate had no obvious effect on the degradation of FPF in ginseng plants. The metabolic behaviour of FPF in ginseng plants may involve cleavage of the –CN group to form DFA and 6-CNA. The production and dissipation of DFA and 6-CNA in ginseng plants are illustrated by the curves in Figure 3. The results indicated that after FPF is degraded in ginseng plants, the main residue is DFA, and the residue of 6-CNA is small. FPF and its two metabolites were metabolised rapidly by ginseng plants. The concentration of FPF metabolites in the ginseng plants was determined by the degradation rate of FPF and its metabolites. The climate difference in different years and regions was the main factor, which mainly affected the degradation rate of FPF and its metabolites by affecting the growth of ginseng (light, temperature, and rainfall). This may be the main reason for the difference in the concentration change of DFA and 6-CNA at Baishan and Yanji in 2018 and 2019.

The concentration of initial deposits of FPF in soil was 0.66–0.91 mg/kg, which dropped to below the LOQ (0.01 mg/kg) after 45 d. The calculated half-lives of FPF in soil were 10.0–16.9 d. Many studies show that microorganisms and organic matter were significant contributors to pesticide degradation in soil [14,15,16]. The soil used to grow ginseng is artificially mixed according to a certain formula, which is typically rich in minerals and organic matter, and treated before planting ginseng. Therefore, the small quantity of microorganisms and the adsorption of organic matter and minerals may explain why FPF has a longer half-life in soil than in ginseng plants. In addition, ginseng plants contain various enzymes [17,18,19,20], and enzyme-catalysed detoxification by ginseng plants might play a dominant role in the rapid degradation of FPF. DFA and 6-CNA were not detected in the soil, probably because of the lower initial FPF deposits in the soil, which cause the concentrations of DFA and 6-CNA in the soil to be lower than the LOQ.

FPF in fresh ginseng did not decompose according to a first-order kinetic relationship. In the dissipation experiment, FPF was sprayed on the surface of the leaves without direct contact with fresh ginseng. Therefore, the residue of FPF in fresh ginseng was accumulated by transport from the leaves and absorption from the soil. The process of transport and absorption is complex and easily affected by the natural environment in the field, resulting in the random change of the FPF residue in fresh ginseng over time.

### 2.4. Terminal Residues of FPF, DFA and 6-CNA in Ginseng Plants, Soil, and Ginseng

The residue definition (for estimation of dietary intake in plant commodities) of FPF was the sum of FPF, DFA, and 6-CNA, and expressed as FPF [11]. Therefore, the total residues were calculated based on molecular weight (Table S5). 

The terminal FPF, DFA, and 6-CNA residues in ginseng plants were detected because ginseng plants may be used for the extraction of saponins (the main medicinal component of ginseng). The concentrations of terminal FPF and 6-CNA residues in ginseng plants were less than the LOQ, and those of the terminal DFA residues were 0.081–0.601 mg/kg (21 d) and 0.096–0.863 mg/kg (28 d). The concentrations of total residues in ginseng plants were 0.272–1.835 mg/kg (21 d) and 0.317–2.623 mg/kg (28 d), which is expressed as FPF.

The concentration of terminal FPF residues in soil was 0.01–0.155 mg/kg (21 d) and 0.01–0.347 mg/kg (28 d), and those of the terminal DFA and 6-CNA residues were lower than the LOQ. The concentration of total residues in the soil was 0.179–0.516 mg/kg (21 d) and 0.179–0.324 mg/kg (28 d), which are expressed as parent equivalents. These data can be used for environmental risk assessments of soil organisms based on the RQ. The RQ was calculated from the predicted environmental concentration (PEC, mg/kg) of the soil and the predicted no-effect concentration (PNEC, mg/kg): RQ = PEC/PNEC. The PNEC was calculated from the toxicity endpoint obtained from ecotoxicological research and the corresponding uncertainty factor (UF): PNEC = endpoint/UF. According to the principle of the maximum risk, the high residue determined in this study was used instead of the PEC. The LC_50_ of FPF for earthworms (acute 14 d) was selected as the toxicity endpoint, and the UF for LC_50_ was 10 [21]. The RQ was <1 (0.019), indicating that the environmental risk of FPF to soil organisms is acceptable. 

The concentrations of terminal FPF, DFA, and 6-CNA residues in fresh ginseng were 0.118–0.436, 0.056–0.110, and 0.013–0.019 mg/kg (21 d); 0.022–0.4, 0.042–0.165, and 0.014–0.018 mg/kg (28 d), respectively. The concentrations of total terminal residues (parent equivalents) were 0.296–0.525 mg/kg (21 d) and 0.228–0.641 mg/kg (28 d). These results were used for the calculation of PFs and dietary risk assessment.

### 2.5. Effect of Processing on Residue Levels in Fresh Ginseng

The PFs were determined from fresh and dried ginseng at intervals of 21 and 28 d; Table 2 shows the PFs of FPF after fresh ginseng was dried. Based on the results, all PFs can be considered comprehensively because the variation is small, and the median value can be used as the best estimate of the PF [22].

In the fresh ginseng processing study, drying increased the concentration of the residues to 0.813–2.363 mg/kg at two locations in 2018 and 2019, with PFs ranging from 3.82 to 4.59 (median). The data were in accordance with those reported by Kim et al. [23], who found that the PFs of difenoconazole in ginseng for drying were 2.00–5.16. Alister et al. [24] reported that more stable pesticides (high hydrolysis DT_50_) were the least reduced during the drying step. FPF is a stable insecticide used for hydrolysis and at high temperature (degradation point is 270 °C) [10]. Therefore, the FPF residues in dried ginseng increased because of water evaporation.

### 2.6. Dietary Rrisk Assessment of FPF in Dried Ginseng

Dried ginseng is typically used in food, health products, and medicine and has gradually become a staple in many countries such as China, Japan, and Korea. With increasing concern from the public over pesticide residues in dried ginseng, various countries such as the USA, EU, Korea, Japan, and China have set MRLs for many pesticides in dried ginseng to protect consumer health. However, the relevant data for FPF are unavailable so far. Therefore, to provide the necessary information for establishing regulations, the dietary risk assessment of FPF in dried ginseng was performed in this study.

The acceptable daily intake (ADI) for FPF established by JMPR was 0–0.08 mg/kg bw [11]. The total national estimated daily intake (NEDI) of FPF were calculated using the STMRs and MRLs (Table 3). The STMRs were obtained from terminal residues experiments in this paper, and the selection of reference MRLs (of the relevant registered crops in China) adhering to the following priority order: China, Codex Alimentarius Commission (CAC), US, Australia, Korea, EU, and Japan [25]. The average body weight of Chinese adults was calculated to be 63 kg [26]. Therefore, the total NEDI (2.0045 mg) was 39.77% of the maximum ADI (5.04 mg) for FPF. The acute reference dose (ARfD) of FPF established by JMPR was 0.2 mg/kg bw [11]. The national estimated short-term intake (NESTI) values of FPF were calculated for dried ginseng using the high residue (estimated in this study) and large portion consumed (obtain from an IESTI calculator was 0.6 g/kg bw/day, available at: https://zwfw.nhc.gov.cn/kzx/tzgg/tzggqb/, accessed on 22 July 2020) (Table 3). The NESTI (1.4478 mg) was 11.49% of the maximum ARfD (12.6 mg) for FPF. The results showed that based on the information provided by this study, the chronic and acute dietary risk of FPF in dried ginseng is acceptable and the long-term and short-term dietary exposures to FPF residues is not a public health risk for typical Chinese consumers.

In addition, the international estimated daily intake (IEDI) and international estimated short-term intake (IESTI) were calculated by the IEDI and IESTI calculator [27] to estimate the dietary exposures of FPF residues to global consumers. The dietary intake data of ginseng in the GEMS/Food regional consumption data (available at: https://extranet.who.int/gemsfood/, accessed on 29 October 2020) were not reported. The Announcement No. 17 (National Health Commission, China, available at: https://zwfw.nhc.gov.cn/kzx/tzgg/tzggqb/, accessed on 11 November 2021) reported that the maximum daily dietary intake did not exceed 3 g/kg bw. Therefore, according to the principle of the maximum dietary risk, this data was used for calculating the IEDI and IESTI. The calculated total IEDIs for the 17 GEMS/Food cluster diets were 0.1–0.4% of the maximum ADI (Table 4). The calculated IESTIs for the 17 GEMS/Food cluster diets of ginseng were 12.07–13.16% of ARfD. The results indicated that the long-term and short-term intake of residues of FPF resulting from its proposed uses is unlikely to present a public health concern for global consumers.

## 3. Materials and Methods

### 3.1. Chemicals and Reagents

The FPF standard (99.5%) and the 17% FPF soluble concentrate were provided by Bayer (Leverkusen, Germany). The 6-CNA (99.2%) and DFA (98.0%) standards were obtained from Chem Service (West Chester, PA, USA). Chromatographic-grade methanol and acetonitrile were purchased from Thermo Fisher Scientific (Waltham, MA, USA). Formic acid was purchased from Sigma-Aldrich (St. Louis, MO, USA). C_18_ and GCB were purchased from Agela Technologies (Tianjin, China). Analytical-grade sodium chloride and anhydrous magnesium sulfate were purchased from Sinopharm Chemical Reagent (Beijing, China). Stock solutions (1000 mg/L) of FPF, 6-CNA, and DFA were prepared in methanol and stored at 4 ± 3 °C (replaced after three months).

### 3.2. Field Experiments

Open field trials on ginseng were carried out from 2018 to 2019 in a mountainous region in Baishan (42°38 N, 126°79 E) and Yanji (42°98 N, 129°49 E) in Jilin Province. The field trials were designed in accordance with the NY/T 788-2018 Guidelines [28]. The sites consisted of treatment plots and control plots of sufficient size to obtain representative samples for each sampling interval (50 m^2^), and each treatment comprised three replicate plots.

For the terminal residue experiments, the 17% FPF soluble concentrate was applied twice at a dosage of 102 g active ingredients per hectare (g a.i./hm^2^) foliar spray. The recommended application interval was 7 d. At least 2.0 kg of soil (at depths of 0–10 cm), 500 g of harvested fresh ginseng samples and 500 g of ginseng plants were randomly collected from 12 points in the test plots at 21 and 28 d after the last application.

For the dissipation experiments, the 17% FPF soluble concentrate was applied once at a dosage of 102 g a.i./hm^2^ foliar spray and soil (no ginseng was planted). At least 2.0 kg of soil (at depths of 0–10 cm), 500 g fresh ginseng, and 500 g ginseng plants were randomly collected from 12 points in the test plots at 0, 1, 3, 7, 14, 21, 28, and 45 d after application.

All samples were placed in sealed sample bags and labelled. The samples were stored at −18 °C before analysis. The storage stability report of JMPR showed that FPF, 6-CNA, and DFA were stable for at least 52 months in high-water, high-acid, high-oil, high-protein, and high-starch-content matrices (representative of plants) when stored in the frozen form at approximately −18 °C [11].

### 3.3. Processing of Fresh Ginseng

Each sample from the terminal residue experiments was divided into two parts for direct analysis and processing procedures. Drying is the most common and simple processing method for fresh ginseng, and the air-drying method is superior to far-infrared and freeze-drying methods [29]. To study the FPF residue in ginseng after processing, fresh ginseng was washed with tap water and dried in a forced air-drying oven (GZX-9070MBE, Shanghai, China) at 50 °C for 10 h. Dried ginseng was cooled at room temperature, sealed, and stored at −18 °C.

### 3.4. Sample Preparation

Processed sample: FPF, DFA and 6-CNA in dried ginseng were analysed by the method developed in our previous study [13].

Raw agricultural commodity (RAC) sample: The fresh ginseng samples were shredded with an electric grinder (FP3010, Braun, Germany), and the ginseng plant samples were crushed using dry ice and an electric grinder before extraction. The prepared fresh ginseng, ginseng plant, and soil samples (10.0 g) were extracted twice with 10 mL of acetonitrile:water (4:1, *v*/*v*) containing 2% formic acid, followed by dilution with water to 25 mL. The diluted extract (1 mL) was purified by the dispersed solid phase extraction method (50 mg of C_18_ for soil and fresh ginseng, 50 mg of C_18_ and 50 mg of GCB for ginseng plant). After centrifugation, the supernatant of the purified solution was filtered using a 0.22 µm syringe filter and analysed by HPLC-MS/MS. 

### 3.5. Instrumental

HPLC-MS/MS was performed using an Agilent 1260-6470 triple quadrupole mass spectrometer (Santa Clara, CA, USA) equipped with an Agilent C_18_ column (3.0 mm × 100 mm, 1.8 µm, ZORBAX RRHD Eclipse Plus). The injection volume was 5 μL. The temperature at both ends of the column was maintained at 30 °C. The mobile phase was a mixture of 0.1% formic acid aqueous solution (phase A) and acetonitrile (phase B). The flow rate was 0.3 mL/min, and gradient elution was carried out as Table S1. The total elution time was 15 min, and FPF and its two metabolites were separated within 10 min.

MS was performed using an electrospray ionisation (ESI) source. FPF was ionised in the positive ion mode, and DFA and 6-CNA were ionised in the negative ion mode. The parameters for the ESI source and for the determination of FPF, DFA, and 6-CNA are listed in Tables S1 and S2, respectively.

### 3.6. Analytical Method Validation

The accuracy (recovery), precision (intraday and interday repeatability), matrix effect, and sensitivity (LOQ) of the method were verified by recovery experiments according to SANTE/11813/2017 [30]. The external standard method was used for the quantification of FPF, 6-CNA, and DFA. Linearity was evaluated by solvent and matrix-matched standard calibration curves (0.001, 0.005, 0.01, 0.05, 0.1, 0.5, and 1 mg/L for FPF and 6-CNA; 0.01, 0.02, 0.05, 0.1, 0.2, 0.5, and 1 mg/L for DFA). The accuracy and precision of the method were determined by fortification experiments that involved spiking blank samples at several levels of FPF (0.01, 0.05, 0.5, and 20 mg/kg), 6-CNA (0.01, 0.05, and 0.5 mg/kg), and DFA (0.05, 0.1, and 1 mg/kg). Add 0.1 mL of the working solution mixture to the blank sample to bring the FPF, DFA and 6-CNA in the blank sample to the respective spiked levels, and then the sample is treated according to the procedure in Section 3.4. Each treatment was performed five times. Precision was expressed as the intraday and interday RSD. The LOQ is defined as the lowest spiked level of the validation, meeting the method performance acceptability criteria. The matrix effect is a common problem that hinders quantitative HPLC-MS/MS analysis (Niessen et al., 2010). At present, the most common methods to compensate for matrix effect include isotope labelling, echo peak technique, extraction solution dilution, and matrix-matching calibration. Matrix-matching calibration was used to obtain more representative results in this study because of its accuracy and convenience, and the matrix effect was calculated using the Equation (1):

Matrix effect (%) = (S_matrix_/S_solvent_ − 1) × 100 (1)

where S_matrix_ is the slope of the matrix-matched calibration curve, and S_solvent_ is the slope of the solvent calibration curve.

### 3.7. Calculation

The degradation kinetics of FPF can be described by a first-order reaction (Equation (2)). When C_t_ = 1/2C_0_, the formula for half-life (t_1/2_, Equation (3)) can be obtained by taking the logarithm on both sides of Equation (2):

C_t_ = C_0_ × e^^(−kt)^ (2)

t_1/2_ = ln2/k (3)

where C_0_ is the initial pesticide residue concentration (mg/kg), C_t_ is the concentration of pesticide residue (mg/kg) at time t (d), and k is the dissipation rate constant.

The PFs were calculated using Equation (4) [31]:

PF = residues (mg/kg) in processed product/residues (mg/kg) in RAC(4)

where RAC is the raw agricultural commodity.

The diet risk assessment is an estimate of the potential residue intake by consumers, including the estimate of both the long-term and short-term dietary exposures. The IEDI and international estimated short-term intake (IESTI) for FPF were calculated for the 17 GEMS/Food cluster diets using the supervised trials median residues (STMRs) and high residues obtained from this paper calculated by an IEDI calculator [27]. The total NEDI and NESTI of FPF were calculated using the Chinese Diet Risk Assessment Model [26]. The risk of pesticide exposure to consumer is acceptable when the estimated dietary intake (per kilogram of body weight) of pesticide residues is less than the ADI or the ARfD [31].

NEDI = ∑(STMR × FI)(5)

NESTI = HR × LP(6)

where FI and LP are the average daily food intake per person (kg/day) and large portion consumed (kg/day).

## 4. Conclusions

In this study, the analytical method, dissipation, terminal residues, processing factor, and dietary risk assessment for FPF and its two metabolites in ginseng plants, soil, fresh ginseng, and its processed products were studied. The method was validated, and satisfactory linearity, repeatability, intermediate precision, and accuracy were obtained. The recoveries were 72.8–97.5%. The method precision was high in terms of repeatability and intermediate precision, with RSD values of 1.1–8.5%. The results of field experiments on dissipation and terminal residues indicated that FPF is an easily degradable pesticide, and it dissipated faster in the ginseng plant (t_1/2_ = 4.5–7.9 d) than soil (t_1/2_ = 10.0–16.9 d). According to the terminal residue study, in which the PFs of FPF in ginseng were studied, the FPF residues in dried ginseng were increased (PF = 3.82–4.59). In addition, chronic and acute dietary risk assessments for FPF in dried ginseng were conducted. The calculated NEDI (2.0045 mg) and NESTI (1.4478 mg) for Chinese consumers were 39.77% of the maximum ADI and 11.49% of ARfD, respectively. The calculated IEDI and IESTI were 0.1–0.4% of the maximum ADI and 12.07–13.16% of ARfD. This study shows that when the recommended dose of FPF was applied to ginseng field, the environmental risk of FPF to soil organisms is acceptable and the harvested fresh ginseng and its products (dried ginseng) would not pose a significant potential risk to global consumers.

## Figures and Tables

**Table 1:** Dissipation kinetics of flupyradifurone in soil and ginseng plants.

Year	Location	Matrix	Regression Equation	Coefficient (R^2^)	Half-Life (d)
2018	Baishan	Soil	C = 0.6854 e^−0.0524t^	0.9777	13.2
		Ginseng plants	C = 16.5064 e^−0.0874t^	0.9884	7.9
	Yanji	Soil	C = 0.6833 e^−0.0409t^	0.9382	16.9
		Ginseng plants	C = 17.0917 e^−0.1428t^	0.9759	4.9
2019	Baishan	Soil	C = 0.6918 e^−0.0693t^	0.9722	10.0
		Ginseng plants	C = 13.3104 e^−0.1522t^	0.9816	4.5
	Yanji	Soil	C = 0.7105 e^−0.0678t^	0.9647	10.2
		Ginseng plants	C = 17.2706 e^−0.1555t^	0.9889	4.5

**Table 2:** Effect of processing on flupyradifurone residues in ginseng.

Year	Location	Processed Fractions	Total Residues (mg kg^−1^)		PFs		Best Estimate
			Pre-Harvest Interval (d)		Pre-Harvest interval (d)		
			21	28	21	28	
2018	Baishan	raw	0.296 ± 0.025	0.228 ± 0.033	/	/	/
		dried	1.277 ± 0.019	0.813 ± 0.028	4.31	3.57	3.94
	Yanji	raw	0.414 ± 0.017	0.641 ± 0.042	/	/	/
		dried	1.634 ± 0.109	2.363 ± 0.027	3.95	3.69	3.82
2019	Baishan	raw	0.461 ± 0.024	0.406 ± 0.019	/	/	/
		dried	2.269 ± 0.092	1.727 ± 0.105	4.92	4.25	4.59
	Yanji	raw	0.525 ± 0.017	0.452 ± 0.033	/	/	/
		dried	2.298 ± 0.024	1.705 ± 0.107	4.38	3.77	4.07

**Table 3:** The long-term and short-term dietary intake risk assessment of flupyradifurone based on the Chinese dietary pattern.

Food Category	FI (kg day^−1^) ^a^	Commodity	MRLs ^b^ (mg kg^−1^)	STMR ^b^ (mg kg^−1^)	HR ^b^ (mg kg^−1^)	Source of Reference Limit
Rice cereals and rice products	0.2399	Rice	3			USA
Wheat cereals and wheat products	0.1385	Maize	0.01			CAC ^d^
Other cereal grains	0.0233	Cereal grains	3			CAC
Potatoes	0.0495	Potato	0.05			CAC
Dried beans and their products	0.016	Beans (dry)	0.4			CAC
Dark-colored vegetables	0.0915	Tomatoes	3			China
Light-colored vegetables	0.1837	Lettuce	4			CAC
Pickles	0.0103					
Fruits	0.0457	Oranges	1			China
Nuts	0.0039	Pecan	0.01			CAC
Livestock and poultries	0.0795	Poultry	0.8			CAC
Milk and milk products	0.0263	Milk	0.7			CAC
Egg and egg products	0.0236	Egg	0.7			CAC
Fish and fish products	0.0301					
Oilseeds and oil	0.0327	Cotton seed	0.8			CAC
Animal origin oil and fat	0.0087	Poultry fat	1			CAC
Sugars and starch	0.0044					
Salt	0.012					
Soy sauce	0.009	Ginseng		1.667 1.801	2.413 2.394	PHI ^c^ of 21 days PHI of 28 days
Total FI (kg day^−1^) ^a^	1.0286					
Total NEDI (mg)	2.0045					
NESTI ^e^ (mg)	1.4478					
ADI (mg/kg bw)	0.08					
ARfD (mg/kg bw)	0.2					
Body weiht (kg bw)	63					
%ADI (%)	39.77%					
%ARfD (%)	11.49%					

^a^ The consumption values of ginseng and other crops referred to the recommended dietary food intake (FI) of an adult (63 kg) per day for its corresponding food classification (data from the dietary guideline published by Health Ministry of the People’s Republic of China). ^b^ The supervised trials median residue (STMR) in ginseng and the maximum residue limits (MRLs) in other crops were used to calculate the national estimated daily intake (NEDI). The high residue (HR) in ginseng was used to calculate the national estimated short-term intake (NESTI). ^c^ PHI: Pre-harvest interval. ^d^ CAC: Codex Alimentarius Commission. ^e^ The large portion consumed of ginseng for calculating the NESTI was 0.6 g/kg bw/day, available at: https://zwfw.nhc.gov.cn/kzx/tzgg/tzggqb/, accessed on 22 July 2020.

**Table 4:** The long-term and short-term dietary intake risk assessment of flupyradifurone based on the Chinese dietary pattern.

Codex Code	Commodity Description	STMR ^a^ (mg/kg)	G01		G02		G03		G04		G05		G06	
			Diet	Intake	Diet	Intake	Diet	Intake	Diet	Intake	Diet	Intake	Diet	Intake
VR 0604	Ginseng, raw	1.801	3.00	5.40	3.00	5.40	3.00	5.40	3.00	5.40	3.00	5.40	3.00	5.40
FS 0013	Subgroup of Cherries, raw	0.555	0.92	0.51	9.15	5.08	0.01	0.01	0.61	0.34	0.06	0.03	6.64	3.69
FS 0014	Subgroup of Plums, raw (including dried plums)	0.23	2.67	0.61	8.77	2.02	0.07	0.02	3.03	0.70	0.70	0.16	4.34	1.00
DF 0014	Plums, dried (prunes)	1.15	0.09	0.10	0.06	0.07	0.01	0.01	0.18	0.21	0.04	0.05	0.06	0.07
FS 2001	Subgroup of peaches, raw (including dried apricots)	0.39	8.01	3.12	5.87	2.29	0.18	0.07	8.19	3.19	1.64	0.64	22.46	8.76
Total intake (ug/person)				9.8		14.9		5.5		9.8		6.3		18.9
Bodyweight per region (kg bw)				60		60		60		60		60		60
ADI (ug/person)				4800		4800		4800		4800		4800		4800
%ADI				0.2%		0.3%		0.1%		0.2%		0.1%		0.4%
**Codex Code**	**Commodity Description**	**STMR ^a^** **(mg/kg)**	**G07**		**G08**		**G09**		**G10**		**G11**		**G12**	
			**Diet**	**Intake**	**Diet**	**Intake**	**Diet**	**Intake**	**Diet**	**Intake**	**Diet**	**Intake**	**Diet**	**Intake**
VR 0604	Ginseng, raw	1.801	3.00	5.40	3.00	5.40	3.00	5.40	3.00	5.40	3.00	5.40	3.00	5.40
FS 0013	Subgroup of Cherries, raw	0.555	1.40	0.78	4.21	2.34	0.04	0.02	2.93	1.63	1.50	0.83	NC	-
FS 0014	Subgroup of Plums, raw (including dried plums)	0.23	5.55	1.28	4.37	1.01	6.08	1.40	3.66	0.84	3.93	0.90	0.46	0.11
DF 0014	Plums, dried (prunes)	1.15	0.61	0.70	0.35	0.40	0.05	0.06	0.35	0.40	0.49	0.56	0.13	0.15
FS 2001	Subgroup of peaches, raw (including dried apricots)	0.39	13.03	5.08	16.29	6.35	8.29	3.23	12.95	5.05	5.35	2.09	0.04	0.02
Total intake (ug/person)				13.2		15.5		10.1		13.3		9.8		5.7
Bodyweight per region (kg bw)				60		60		55		60		60		60
ADI (ug/person)				4800		4800		4400		4800		4800		4800
%ADI				0.3%		0.3%		0.2%		0.3%		0.2%		0.1%
**Codex Code**	**Commodity Description**	**STMR^a^** **(mg/kg)**	**G13**		**G14**		**G15**		**G16**		**G17**			
			**Diet**	**Intake**	**Diet**	**Intake**	**Diet**	**Intake**	**Diet**	**Intake**	**Diet**	**Intake**		
VR 0604	Ginseng, raw	1.801	3.00	5.40	3.00	5.40	3.00	5.40	3.00	5.40	3.00	5.40		
FS 0013	Subgroup of Cherries, raw	0.555	0.01	0.01	0.01	0.01	5.96	3.31	0.01	0.01	NC	-		
FS 0014	Subgroup of Plums, raw (including dried plums)	0.23	0.07	0.02	0.02	0.00	16.65	3.83	0.01	0.00	NC	-		
DF 0014	Plums, dried (prunes)	1.15	0.01	0.01	0.01	0.01	0.37	0.43	0.01	0.01	NC	-		
FS 2001	Subgroup of peaches, raw (including dried apricots)	0.39	0.02	0.01	0.01	0.00	10.76	4.20	0.01	0.00	NC	-		
Total intake (ug/person)				5.4		5.4		17.2		5.4		5.4		
Bodyweight per region (kg bw)				60		60		60		60		60		
ADI (ug/person)				4800		4800		4800		4800		4800		
%ADI				0.1%		0.1%		0.4%		0.1%		0.1%		

^a^ The supervised trials median residue (STMR) in ginseng were obtained from the terminal residues experiments in this paper and in other crops were obtained from the JMPR report 2017 (https://www.fao.org/agriculture/crops/thematic-sitemap/theme/pests/lpe/lpe-f/en/, accessed on 15 July 2021).

## Data Availability

Not applicable.

## Supplementary Materials

The following supporting information can be downloaded at: https://www.mdpi.com/article/10.3390/molecules27175473/s1, Figure S1: Chemical structure of flupyradifurone; Table S1: The MRM and gradient elution conditions for analysis of flupyradifurone, DFA and 6-CNA; Table S2: The parameters for the ESI source in HPLC-MS/MS; Table S3: Mean recoveries and RSD for target compounds from different matrices at three spiked levels; Table S4: Calibration information of FPF, 6-CNA, or DFA in different matrices; Table S5: Terminal residues of FPF, 6-CNA and DFA in soil, ginseng, and ginseng plants (*n* = 3).

## Sample Availability

Samples of the compounds are not available from the authors.
