# Supplementary material for: Dissipation, Processing Factors and Dietary Risk Assessment for Flupyradifurone Residues in Ginseng
Source: Molecules. 2022 Aug 26;27(17):5473. doi: 10.3390/molecules27175473 (PMC9457792; doi:10.3390/molecules27175473)
Supplement: Supplementary file 1 [file molecules-27-05473-s001.zip › molecules-1868451-supplementary.pdf]

Supplementary materials

Dissipation, Processing Factors and Dietary Risk Assessment for Flupyradifurone Residues in Ginseng

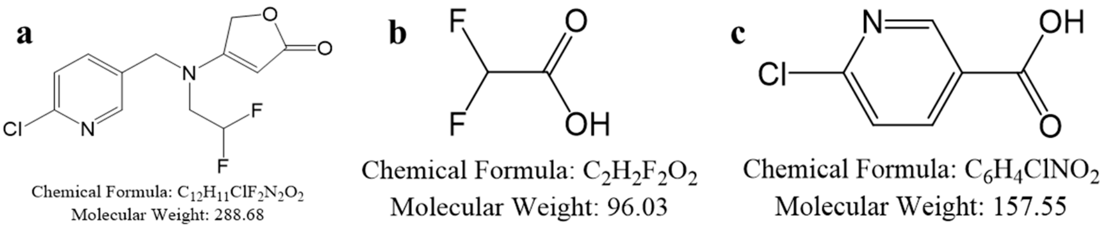

Figure S1. Chemical structure of flupyradifurone (FPF, a), difluoroacetic acid (DFA, b) and 6-chloronicotinic acid (6-CNA, c).

Table S1. The MRM and gradient elution conditions for analysis of FPF, DFA and 6-CNA.

Compound	Ion source	Retention time	Precursor ion	Quantification ion (CE[a])	Confirmation ion (CE[a])	Fragmentor	Gradient elution	
							Time (min)	Phase A (%)
FPF	ESI+	5.84	289.0	245.1 (15)	126.1 (25)	140	0	95
							2	70
DFA	ESI-	0.82	95.2	51.1 (10)	51.1 (10)	70	4	60
							5	40
6-CNA	ESI-	2.64	156.0	111.9 (8)	35.1 (10)	40	6-9	20
							9-15	95

[a] collision energy (eV), it is given in parentheses.

Table S2. The parameters for the ESI source in HPLC-MS/MS.

**Parameters**	**Positive mode**	**Negative mode**
Nebulizer pressure (psi)	30	30
Capillary voltage (kV)	4.0	4.0
Nozzle voltage (kV)	0.5	0.5
Desolvation flow rates[a] (L/min)	7	7
Sheath gas flow rates (L/min)	8	8
Desolvation temperature (°C)	330	330
Source temperature (°C)	300	300

[a] Desolvation (L/min) is the drying gas.

Table S3. Mean recoveries and RSD for target compounds from different matrices at three spiked levels.

Compound	Intraday (n=5) Average recovery, % (RSD_r[a], %)										Inter-day (n=15) Average recovery, % (RSD_r[a], %)		
	DFA			6-CNA			FPF				DFA	6-CNA	FPF
Spiked level (mg kg^{-1})	0.05	0.5	1	0.01	0.05	0.5	0.01	0.05	0.5	20	0.5	0.05	0.05
Ginseng, dry	90.3 (2.1)	86.1 (1.8)	88.2 (2.8)	93.7 (3.2)	92.3 (4.4)	93.6 (2.8)	96.7 (3.7)	93.5 (2.5)	94.1 (4.5)	92.8 (3.6)	81.4 (5.6)	88.9 (7.1)	93.2 (3.7)
Ginseng	73.4 (3.1)	78.5 (3.8)	74.2 (1.6)	83.5 (2.2)	81.7 (2.5)	82.6 (2.4)	95.3 (2.1)	92.5 (1.8)	94.1 (1.5)	93.4 (1.8)	75.6 (7.1)	76.8 (6.9)	90.4 (4.2)
Ginseng plant	72.8 (2.4)	74.9 (2.7)	77.3 (5.2)	80.3 (1.1)	81.1 (1.6)	82.8 (5.7)	93.7 (4.2)	91.5 (2.7)	94.8 (2.1)	91.6 (1.6)	76.8 (8.4)	77.4 (8.5)	91.2 (5.5)

Soil	84.7 (3.7)	85.1 (2.6)	81.2 (3.8)	82.6 (1.8)	88.0 (4.1)	85.4 (3.9)	94.8 (2.1)	93.1 (2.9)	95.7 (3.3)	95.9 (2.9)	78.2 (7.8)	79.6 (8.2)	97.5 (3.3)

[a] The intraday relative standard deviation for repeatability (n=5).

[b] The inter-day relative standard deviation for repeatability (n=15).

Table S4. Calibration information of FPF, 6-CNA, or DFA in different matrices.

Compound	Matrix	Calibration equation	R^2	ME (%)	LOQ (mg kg^{-1})
DFA	Ginseng, dry	y = 190525.0731 x - 472.7148	0.9998	-17.93	0.05
	Ginseng	y = 73395.5776 x - 125.1053	0.9993	-45.62	0.05
	Ginseng plants	y = 37248.2556 x - 308.1053	0.9990	-49.25	0.05
	Soil	y = 134967.9618 x - 1338.4737	0.9996	-29.16	0.05
6-CNA	Ginseng, dry	y = 1220180.3372 x - 3451.1053	0.9999	-19.98	0.01
	Ginseng	y = 1016364.6838 x - 2764.2105	0.9992	-32.63	0.01
	Ginseng plants	y = 651997.9447 x - 3722.6842	0.9997	-35.85	0.01
	Soil	y = 1508630.9690 x - 12693.5789	0.9991	23.64	0.01
FPF	Ginseng, dry	y = 4742781.5132 x - 104946.3684	0.9999	-23.34	0.01
	Ginseng	y = 3578219.6848 x - 16563.8947	0.9996	-30.42	0.01
	Ginseng plants	y = 5065327.7858 x - 58618.4211	0.9995	-41.56	0.01
	Soil	y = 5142597.9948 x - 121450.4737	0.9991	8.43	0.01

Table S5. Terminal residues of FPF, 6-CNA and DFA in soil, ginseng, and ginseng plants (n = 3).

Matrix	Year	Location	PHI[a] (d)	Residues (Mean value ± standard deviation, mg kg^{-1})			
				FPF	DFA	6-CNA	Total residues[b]
Soil	2018	Baishan	21	0.231 ± 0.012	< 0.050	< 0.010	0.400 ± 0.012
			28	0.153 ± 0.015	< 0.050	< 0.010	0.322 ± 0.015
		Yanji	21	< 0.010	< 0.050	< 0.010	< 0.179
			28	< 0.010	< 0.050	< 0.010	< 0.179
	2019	Baishan	21	0.200 ± 0.011	< 0.050	< 0.010	0.369 ± 0.011
			28	0.116 ± 0.006	< 0.050	< 0.010	0.285 ± 0.006
		Yanji	21	0.347 ± 0.009	< 0.050	< 0.010	0.516 ± 0.009
			28	0.155 ± 0.010	< 0.050	< 0.010	0.324 ± 0.010
Ginseng plants	2018	Baishan	21	< 0.010	0.601 ± 0.008	< 0.010	1.835 ± 0.029
			28	< 0.010	0.863 ± 0.016	< 0.010	2.623 ± 0.036
		Yanji	21	< 0.010	0.498 ± 0.014	< 0.010	1.525 ± 0.034
			28	< 0.010	0.352 ± 0.007	< 0.010	1.086 ± 0.027
	2019	Baishan	21	< 0.010	0.081 ± 0.001	< 0.010	0.272 ± 0.019
			28	< 0.010	0.096 ± 0.003	< 0.010	0.317 ± 0.022
		Yanji	21	< 0.010	0.154 ± 0.006	< 0.010	0.491 ± 0.026
			28	< 0.010	0.281 ± 0.009	< 0.010	0.873 ± 0.029
Ginseng, dried	2018	Baishan	21	0.318 ± 0.012	0.274 ± 0.007	0.074 ± 0.008	1.277 ± 0.019
			28	0.052 ± 0.007	0.230 ± 0.010	0.038 ± 0.009	0.813 ± 0.028
		Yanji	21	0.436 ± 0.056	0.362 ± 0.012	0.060 ± 0.010	1.634 ± 0.109
			28	0.190 ± 0.017	0.684 ± 0.009	0.064 ± 0.009	2.363 ± 0.027
	2019	Baishan	21	1.586 ± 0.117	0.180 ± 0.011	0.058 ± 0.005	2.269 ± 0.092
			28	1.098 ± 0.093	0.192 ± 0.010	0.048 ± 0.010	1.727 ± 0.105
		Yanji	21	1.600 ± 0.012	0.180 ± 0.007	0.056 ± 0.002	2.298 ± 0.024
			28	1.080 ± 0.097	0.198 ± 0.013	0.046 ± 0.008	1.705 ± 0.107
Ginseng, raw	2018	Baishan	21	0.140 ± 0.009	0.085 ± 0.024	0.018 ± 0.003	0.296 ± 0.025
			28	0.022 ± 0.011	0.071 ± 0.014	0.014 ± 0.002	0.228 ± 0.033
		Yanji	21	0.118 ± 0.034	0.110 ± 0.018	0.019 ± 0.004	0.414 ± 0.017
			28	0.053 ± 0.019	0.165 ± 0.013	0.018 ± 0.002	0.641 ± 0.042

2019	Baishan	21	0.436 ± 0.023	0.065 ± 0.012	0.013 ± 0.003	0.461 ± 0.024
		28	0.238 ± 0.015	0.050 ± 0.007	0.014 ± 0.004	0.406 ± 0.019
	Yanji	21	0.355 ± 0.018	0.056 ± 0.004	0.017 ± 0.003	0.525 ± 0.017
		28	0.400 ± 0.044	0.042 ± 0.003	0.014 ± 0.003	0.452 ± 0.033

[a] PHI: Pre-harvest interval.

[b] The sum of FPF, DFA and 6-CNA, expressed as parent equivalents.
